# Power Approaches for Implantable Medical Devices

**DOI:** 10.3390/s151128889

**Published:** 2015-11-13

**Authors:** Achraf Ben Amar, Ammar B. Kouki, Hung Cao

**Affiliations:** 1 LACIME Laboratory, University of Quebec, ÉTS, 1100 Notre-Dame West, Montreal, QC H3C 1K3, Canada; E-Mail: ammar.kouki@etsmtl.ca; 2 Division of Engineering, STEM, University of Washington, Bothell, WA 98011, USA; E-Mail: hungcao@uw.edu

**Keywords:** implantable medical devices, energy harvesting, wireless power transfer, power management, inductive coupling

## Abstract

Implantable medical devices have been implemented to provide treatment and to assess *in vivo* physiological information in humans as well as animal models for medical diagnosis and prognosis, therapeutic applications and biological science studies. The advances of micro/nanotechnology dovetailed with novel biomaterials have further enhanced biocompatibility, sensitivity, longevity and reliability in newly-emerged low-cost and compact devices. Close-loop systems with both sensing and treatment functions have also been developed to provide point-of-care and personalized medicine. Nevertheless, one of the remaining challenges is whether power can be supplied sufficiently and continuously for the operation of the entire system. This issue is becoming more and more critical to the increasing need of power for wireless communication in implanted devices towards the future healthcare infrastructure, namely mobile health (m-Health). In this review paper, methodologies to transfer and harvest energy in implantable medical devices are introduced and discussed to highlight the uses and significances of various potential power sources.

## 1. Introduction

In the past few decades, we have witnessed tremendous development in electronics, micro- and nano-fabrication, and wireless technology which have greatly enhanced the quality and efficacy of healthcare as well as life-science research [1,2,3,4]. These innovations dovetailed with advanced biomaterials have enabled miniaturized sensors and biocompatible devices that could be implanted *in vivo* in humans and animal models, allowing diagnosis, prognosis and biological investigations [5,6,7]. Chronic diseases of inner organs have been elucidated in animal models, and/or diagnosed and treated in real patients with support from numerous implantable devices [6,8,9,10,11]. Recently, flexible and stretchable electronics has been introduced and demonstrated, showing promise for the future of healthcare, biological-science discoveries and medicine [2,7,12]. Unreachable locations in vertebrates’ bodies challenging scientists such as the deep brain, intravascular regions, inside the heart or even a location inside a single cell have been assessed and investigated with miniaturized implantable systems [13,14,15,16].

Implantable Medical Devices (IMDs) to improve healthcare, aiding or delivering the functions of certain malfunctioning organs have been around for years. They have been utilized for diagnosis, prognosis and treatment. IMDs can be categorized as active and passive devices depending on whether they require a power source or not, respectively. Nowadays, a host of chronic diseases have been addressed using IMDs all over the body, from the brain, cochlea and retina to the heart, lungs, knee joints and even vessels, the esophagus and the bladder. According to statistics from a decade ago, there were about three million people around the world with pacemakers and each year 600,000 more pacemakers were being implanted. Besides, more than 60,000 people were treated with cochlear implants [17]. The numbers have been rapidly increasing in recent years due to the larger population and a better healthcare system. For example, more than 230,000 new pacemakers were implanted in the USA in 2009 [18]. These facts indicate that IMDs have become more and more popular in humans’ life.

To ensure proper operation, most IMDs need to rely on a permanent and sufficient power supply, thus numerous power sources for IMD have been widely investigated in the last decades. Different power approaches can allow for the autonomous operation of IMDs by generating electrical power to replace or supplement existing battery power. The existing major challenges have been size limitations, inaccessibility, the need to work continuously and biocompatibility.

Since the first medical implant, the pacemaker, was introduced in 1972 using batteries [19,20,21,22,23], various types of batteries have been developed and deployed for IMDs [24,25,26,27,28,29,30,31,32,33]. Among those, lithium-based (Li) batteries have been the most popular power source owing to their high volumetric energy density as well as comparatively compact sizes [28,34,35]. Further, they have a considerably durable longevity of 5 to 10 years, and thus are appropriate for long-term applications [36]. Besides, bio-fuel cells exploiting biocatalysts for generating electric power from renewable biodegradable materials such as glucose or amylum are also potential sources [30,35]. Enzyme-based biofuel cells are able to operate under mild conditions (20–40 °C and close to pH = 7.0) generating milliwatt level power. Making them suitable for the majority of IMDs such as pacemakers, cardiac defibrillators and drug delivery systems. One other type—nuclear batteries—which was also introduced in early 1970s, were utilized to extend the lifespan of IMDs for more than 10 years. Their operation is based on the carried energy emitted by the particles from radioisotopes [37]. However, they were discontinued in the 1980s due to the potential risks and the conclusion from physicians that IMDs should be updated with new technologies at least once a decade [38].

Alternative solutions have been also proposed, investigated and developed, by which energy was generated and harvested from potential sources surrounding the implants. The aforementioned biofuel cells are great candidates since they can exploit glucose and oxygen which are abundant in the blood to generate energy [27,39,40,41]. Further, vertebrates’ bodies and their daily activities are great sources of energy through heating (body heat) and movements like breathing and motion, that could be exploited to power up IMDs replacing the traditional batteries. For instance, the maximum temperature difference between the inner parts and the skin is 8 °C, which is sufficient to generate a few hundred microwatts of electricity using thermoelectric generators [42,43,44]. On the other hand, piezoelectric generators have been employed to convert kinetic energy into electricity utilizing piezoelectric materials [45,46,47]. Although they are able to generate considerably higher power, on the order of milliwatts, piezoelectric generators usually require massive motion, and hence are not appropriate for most implant locations [48,49,50]. Electrostatic and electromagnetic mechanisms have been also used to harvest energy using body motions, however their size limitations and low generated power limits their use in practical *in vivo* applications [51,52,53,54].

**Figure 1:**
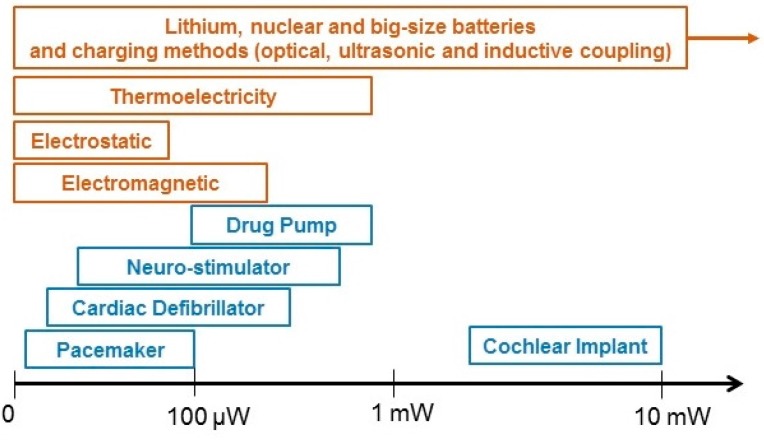
Power ranges of methods and IMDs. The scale is not in the right ratio, it is only for a conceptual illustration. Batteries and charging methods can deliver high power; however, in practical scenarios, it depends on the location of IMD, the size of IMD as well as the tissue.

Instead of exploiting potential sources generated by the host, energy could be supplied to IMDs by an external unit to either charge the battery or continuously power the batteryless implant. This may be accomplished optically, ultrasonically or electromagnetically. Optical-charging methods consist of a photovoltaic cell in the IMD which receives power from a laser diode usually operating in the near-infrared or infrared range [55]. Ultrasonic devices have attracted growing interest in recent years due to their comparative efficiency, compactness and immunity to electromagnetic radiation [56,57,58,59,60]. Nonetheless, inductive power transmission across the body tissue is currently the only viable solution to deliver sufficient power to various kinds of IMDs with miniaturized dimensions [61]. This method is based on a pair of antennas by which power could be transferred through a mutual inductive coupling link. Therefore, antenna design and orientation, working distance and frequency, as well as the designated power would be considered. The power ranges of all the methods and IMDs are summarized in Figure 1.

**Figure 2:**
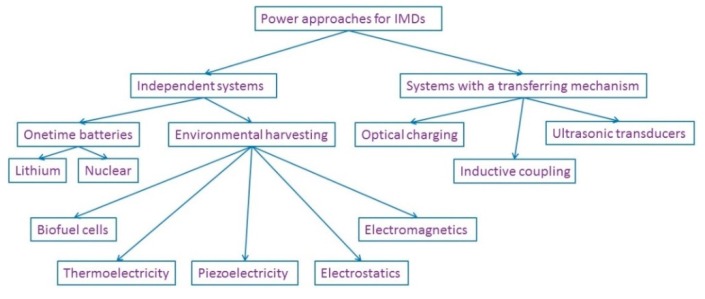
Approaches to power IMDs.

In this review paper, we introduce methods to power active IMDs and present insightful discussions about each method. Approaches are categorized into two main groups: (1) IMDs that work independently with or without a one-time battery, or sustainably; and (2) IMDs which could be either battery-based or batteryless, obtaining power transferred from an external unit. Figure 2 presents these two groups. An overview about ultimate goals of IMDs is presented in the Discussion section followed by in-depth discussions of the most popular method, namely inductive coupling.

## 2. Methods to Power IMDs

### 2.1. Independent Systems

The first battery was invented by Volta in 1796 and since then numerous types have been discovered and applied to diverse uses in human life [62]. In general, batteries store energy in the forms of chemical substances which can produce electricity. Batteries contain anodes, cathodes and electrolytes to allow ions to move thus forming currents. There are three categories of power capabilities used for battery performance (low rate, medium rate and high rate). Although the definition is not yet clear, low-rate batteries should be able to provide a constant current of 100 µA while high-rate ones can supply a pulse power of at least 5 W for 10 s. Thus, the gap could be filled with medium-rate batteries. Among IMDs, pacemakers use low currents, neuro-stimulators and drug pumps need medium-rate ones and implantable defibrillators/cardioverters (ICDs) require extremely high power as well as additional longevity [32].

#### 2.1.1. Lithium Batteries

Typically, Li batteries were developed and appeared in the forms of Li metal anodes with cathode systems including iodine (Li/I_2_) [2,6,24,63,64], manganese oxide (Li/MnO_2_) [7,12,65,66], carbon mono fluoride (Li/CF_x_) [67,68,69,70], silver vanadium oxide (Li/SVO) [71,72,73] or hybrid cathodes (Li/CF_x_-SVO) [29,32,64,74,75]. As reliable sources for long-term applications such as cochlear implants, pacemakers, cardiac defibrillators or drug delivery, these Li batteries have been widely employed to provide appropriate power levels ranging from microamperes to amperes, as demanded by different types of IMDs [26,29,32].

Among those, Li/I_2_ batteries have been proved to be safer and more reliable than others for uses with implantable pacemakers, thus they have been utilized by most manufacturers during the last 40 years [27]. Li/I_2_ batteries have a discharge voltage of up to 3.6 V, which is equal to three times the voltage generated by Ni-based cells. Their energy density can reach 210 W·h/kg, which can power a cardiac pacemaker for several years. Further, it is usually easier to measure the remaining energy of Li/I_2_ batteries than other Li-based batteries, thus providing enough time to change the battery for IMDs [27]. On the other hand, Li/SVO batteries have been employed majorly for ICDs in the last 30 years owing to their high capacity of over 300 W·h/kg. The presence of metallic silver also drastically improves the electronic conductivity [76]. In the mid-range, the systems with cathodes of thionyl-chloride (Li/SOCl_2_) have been widely used for neuro-stimulators and drug pumps.

The recent advances in material research have led to a new class of Li-based batteries, namely flexible Li or Li polymer batteries [77,78]. While its advantages in flexible electronics and wearable consuming devices are undoubted, further implementations for implants have been limited due to the remaining obstacles in size and questions related to potential toxicity.

#### 2.1.2. Bio-Fuel Cells

**Figure 3:**
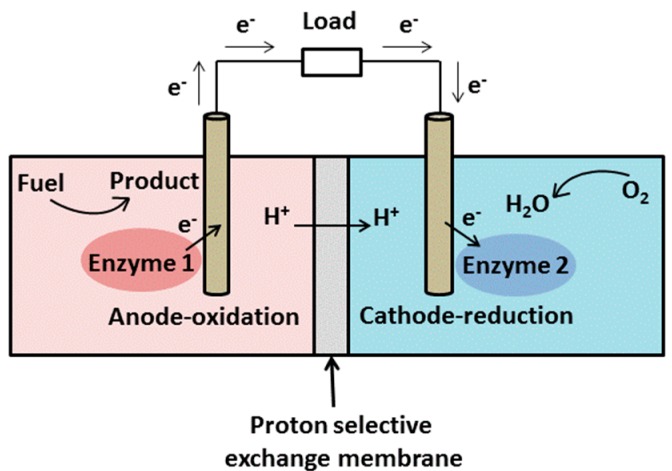
Schematic of a bio-fuel cell.

In general, bio-fuel cells are devices that transform biochemical energy into electricity based on electrochemical reactions involving biochemical pathways [30]. The link between biology and electricity was first discovered by Galvani in his experiments with frogs’ legs in 1791. He found the muscles of dead frogs’ legs twitched when electrical sparks were applied. The concept of ‘fuel cell’ was demonstrated in 1839 when Grove fully reversed the electrolysis process of water. He was able to recombine oxygen and hydrogen to produce water and interestingly, an electrical current [79]. The development of microbial-fuel cells (MFC) was then pioneered by Potter in 1911 when he discovered that one could generate a current of 0.2–0.5 mA at 0.3–0.5 V when a platinum electrode was placed into cultures of E. coli [80]. This indicated the potential of using microorganisms to generate power. The demonstration was achieved in 1931 when Cohen implemented biofuel cells providing 2 mA of current and 35 V voltage [30,81]. In a fuel cell, there are oxidation and reduction reactions occurring at the anode and cathode, respectively. Electrons released by the oxidation travel to the cathode to form an electrical current via an external circuit [30]. At the same time, protons move to the cathode through a proton-selective exchange membrane (Figure 3) [27].

Based on the catalyst used, bio-fuel cells can be classified into microbial fuel (MFC) or enzymatic bio-fuel (EBC) cells; whereas considering the pathway through which electrons move from anode to cathode, we can distinguish between direct or non-direct cells [27,30]. For example, the fuel can come from glucose and oxygen in the blood following these reactions:

At anode: C_6_H_12_O_6_ → C_6_H_10_O_6_ + 2H^+^ + 2e^−^

At cathode: O_2_ + 4H^+^ + 4e^−^ → 2H_2_O

The interest in MFCs was triggered in 1960s owing to the USA space program during which possible powering technologies had been sought for a waste disposal system for space flights [30]. The application of MFCs for IMDs was first initiated in 1960s when cell-free enzyme-based fuel cells were used for implantable artificial hearts [30,82]. During the 1970s, EBCs using glucose as fuel and oxygen as oxidizer were investigated to provide power for IMDs [27,30] and since then, various approaches and modifications have been proposed to enhance the performance as well as to target specific applications [28]. In 2003, Mano and colleagues reported a miniaturized bio-fuel cell capable of operation while implanted in a grape. They were able to produce a power of 2.4 μW at 0.52 V which was very promising for the applications with compact implants [83].

Bio-fuel cells present several advantages including the use of existing recyclable materials in Nature, the moderate operating conditions for the reactions and the biocompatibility between bio-fuel cells and the human body [31]. Nevertheless, challenges still remain. Firstly, it is difficult to maintain the biocatalyst over a long period and surgical intervention is of course not a desired option. Secondly, the microwatt level of bio-fuel cells limits their use in a wide range of applications. Finally, even though they are highly biocompatible, unavoidable biofouling still occurs that can damage the device or harm the patients [27].

#### 2.1.3. Nuclear Batteries

In nuclear batteries, power is transformed to electricity through energy carried by particles emitted from radioisotopes. This process could be achieved in several ways. For instance, some utilize the electric potential difference produced by the particle emitted from the radioisotopes, some use the electric potential coming from the ionization of emitted particle bundles, others employ the photoelectrical conversion prompted by a fluorescent material, and the rest exploit the heat energy of radiation. The advantages of nuclear batteries lie in the fact that they can provide much longer service life (>15 years) than all other competitors, and their output energy is extremely stable, regardless of environmental factors (temperature, pressure and electric field). For example, the Betacel produced by Medtronics, Inc. (Minneapolis, MN, USA) with a volume of 1.8 mL, a height of 1.02 cm and a diameter of 1.52 cm, can provide 50 μW of power [27]. Further, their safety has been proved for as long as they are kept hermetic, however, the potential radioactivity danger as well as their expensive cost make them still unacceptable [32,33].

#### 2.1.4. Thermoelectricity

**Figure 4:**
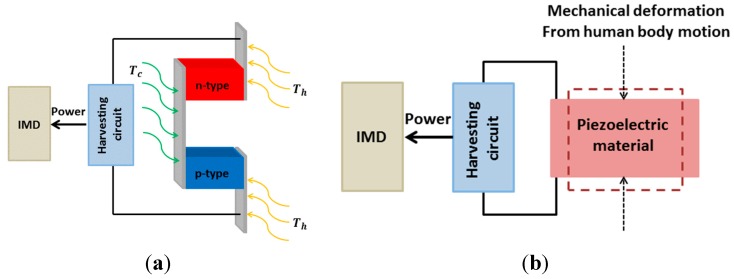
Conceptual views of (**a**) thermoelectricity; and (**b**) piezoelectricity.

The thermoelectric effect could be exploited to obtain electrical energy using the temperature differences in the human body. Naturally, our body presents temperature differences between locations thus forming temperature gradients. When a temperature gradient is applied in a thermoelectric module, a potential difference appears across the material through the Seebeck effect (Figure 4a) [84]. Since the human body is an unlimited heat-energy source, these generators’ lifetime is also naturally unlimited [85,86,87,88]. Nevertheless, the thermal gradients in the human body are relatively small, making it difficult to achieve significant power outputs. Since thermoelectric generators are a type of heat engine, their efficiency is proportional to the Carnot efficiency which is defined as [89]:

(1) $${\text{ŋ}}_{c}=1-\frac{T_{c}}{T_{h}}=\;\frac{T_{h}-\;T_{c}}{T_{h}}$$

where ŋ_c_ is the Carnot efficiency, *T_h_* and *T_c_* represent the hot and cold temperatures in Kelvin, respectively. TEGs are normally made up of semiconductor materials, with the most common ones being bismuth telluride (Bi_2_Te_3_) and polycrystalline silicon-germanium (poly-SiGe) film [90]. A thermoelectric module is usually formed by n-doped and p-doped semiconductor thermocouples placed electrically in series and thermally in parallel. In 1999, Stark and Stordeur reported a 0.19 cm^3^ thermoelectric generator which could produce 1.5 µW and 5.8 µW at 5 K and 10 K of temperature gradient, respectively [91]. The device was fabricated by sputtering the thermoelectrically effective material Bi_2_Te_3_ on 75 µm thick Kapton (Dupont, Wilmington, DE, USA) substrates to obtain the thermopile structure. The final integration density was 11.9 thermocouples/mm^3^. Later in 2004s, Strasser *et al.* introduced their work in which micro-machined CMOS thermoelectric generators were developed using poly-SiGe material deposited on a polysilicon substrate. They obtained a power output of 1 µW with generators of 1 cm^2^ in size with respect to 5K in temperature gradient [92]. In fact, the maximum temperature difference between the inner part of the body and the skin surface is about 8 K, therefore, the maximum output power can be about 180 μW/cm^2^ [93]. Thus it can be seen that thermoelectric generators only provide sufficient power for IMDs which require low microwatt power. To increase the output power, a large number of thermocouples needs to be cascaded in a proper way but this would face the issues of size, reliability and biocompatibility [84].

#### 2.1.5. Piezoelectricity

The piezoelectric phenomenon was discovered in 1880 by the French physicists Jacques and Pierre Curie [94]. It could be verified that certain materials generate a proportional electrical polarization with respect to the applied mechanical stress via the piezoelectric effect. To date, numerous piezoelectric materials have been utilized to generate energy for various applications [95,96]. The physical phenomenon underlying here is based on the fact that the electrical charge accumulating in certain solid materials will be induced when it is subject to mechanical deformation (Figure 4b). Therefore, piezoelectric transducers could exploit the mechanical energy produced by our body motions [45,46,47]. The human body motions can be classified into two categories: continuous motions such as human respiration and blood flow, and discontinuous motions such as walking and hand movement. Several groups have investigated the use of discontinuous human body motion for piezoelectric energy harvesting [97,98,99]. The piezoelectric transducer is integrated in moving locations such as joints, whole-body center mass motion, muscle twitches and into shoes [98,100,101,102,103]. The ankle, knee, hip, elbow, and shoulder motions can generate up to 69.8 W, 49.5 W, 39.2 W, 2.1 W, and 2.2 W, respectively [98]. On the other hand, the continuous motions obtained from blood flow or respiration can provide lower power compared with that of the piezoelectric power harvester using discontinuous motion. For example, the maximum power generated by a piezoelectric plate with a radius of 5.62 mm and a thickness of 28 μm in a blood-flow environment is 0.33 μW [104]. Obviously, piezoelectric harvesters are good candidates to power implants, but they need significant movements to generate considerable power, thus the practical implantable locations are limited (*i.e.*, knee, elbow, foot). For instance, efforts by Kymissis *et al.* in 1998 resulted in a 1 W of output power delivered by a piezoelectric transducer integrated in a shoe heel [46]. However, achieving and harvesting energy from such a system *in vivo* remain the most important questions to answer. Again, the small size and the biocompatibility issues are always the biggest challenges.

#### 2.1.6. Electrostatic Generators

**Figure 5:**
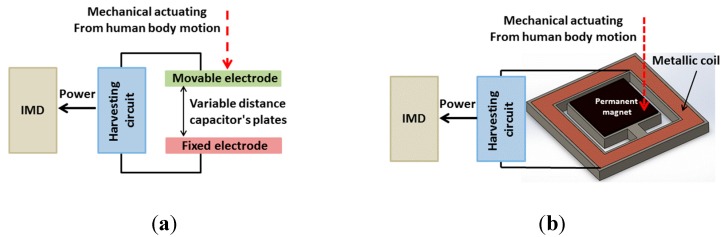
Conceptual views of (**a**) electrostatic; and (**b**) electromagnetic generators.

Electrostatic generators produce electricity via electrostatic induction which is based on the electrostatic potential energy as the result of conservative Coulomb forces [105]. They are designed to exploit mechanical motion to induce electrical energy as the moving parts of the transducer are under effect of an electric field. A common construction for electrostatic generators consists of two conductive plates that are electrically isolated via air, vacuum or a dielectric insulator (capacitor), and relatively mobile (Figure 5a) [53]. The distance between the two electrodes of the capacitor changes due to the movement or to the vibration of one movable electrode caused by the motion of human body.

There are two operation modes for an electrostatic energy harvester: constant charge mode and constant voltage mode [106]. The plates are charged via an external source such as a battery. Besides, there are three different types of electrostatic generators which can be defined according to their actuation direction: the in-plane gap closing, out-of-plane closing and in-plane overlap [107]. The in-plane gap closing techniques offer the highest output power compared to other actuation techniques. Electrostatic generator harvesting systems can be built by silicon micromachining fabrication techniques and present good integration capabilities with microelectronic circuits and other technologies, thus standing out as good candidates for IMDs. Microelectromechanical systems-based (MEMS-based) technologies enable miniaturized size, making these generators implantable inside the human body [52,108]. Recently, several works on the use electrostatic power harvesting systems to power biomedical devices have been reported. For example, an electrostatic generator was reported to exploit ventricular motion and heartbeat to feed a cardiac pacemaker and could generate 36 μW and 58 μW, respectively [54,109]. Further, Meninger *et al.* developed an electrostatic generation system which could produce 8 µW of power using MEMS-based technology. In their work, Miao and colleagues implemented a non-resonant MEMS-based electrostatic generator, which could thus provide flexibility of operation with a wide range of excitation frequencies. They obtained 80 µW for operation at 30 Hz with respect to movement of 0.1 m/s [108]. Overall, the high output impedance and voltage of the electrostatic generators making them less suitable for power supply devices due to the reduced amounts of available current due to the high output voltage. In addition, the amount of energy produced is typically low and these generators usually need an additional voltage source to initially charge the capacitor, representing the major disadvantage of this technology.

#### 2.1.7. Electromagnetic Generators

Electromagnetic generators harvest energy based on the Faraday–Neumann–Lenz law which states that the relative motion between a coil and a permanent magnet produces a time-variable magnetic flux and consequently generates a voltage. This can be done in two ways: (1) relative motion is utilized while the generating system is fixed; and (2) rigid body motion is used with the inertia force of a weight on the generator [110]. Typically, the power is produced through the relative movement of the magnet and coil, or due to the changes in the magnetic field [51,111]. Therefore, the amount of electricity generated can be a function of magnetic field strength, relative motion velocity and the number of turns of the coil. Like those approaches based on piezoelectric or electrostatic transducers, electromagnetic generators exploit human body motions to generate power (Figure 5b).

The low-frequency and irregular movements of humans make electromagnetic techniques suitable for powering IMDs. For example, with the repeating contraction of the heart muscle at a frequency between 0.5 and 2 Hz, a power of 40–200 μW could be generated [112,113]. Roberts *et al.* investigated an electromagnetic generator using MEMS technology to enhance the power for pacemaker batteries in clinical trials [114]. The abdomen moves with a frequency of 0.3 Hz during breathing, producing a power of about 1.1 mW through an electromagnetic generator with a volume of 16 cm^3^ [115]. Using human walking, an electromagnetic generator with a size of 2.6 mm^2^ could generate 400 μW [116]. High performance bulk magnets, multi-turn and macroscale coils are readily available; nevertheless, the main challenge of the MEMS fabrication technology utilized in this approach is the poor properties of planar magnets [51]. In addition, the electromagnetic generators produce lower power compared with that obtained by piezoelectric generators.

### 2.2. Systems with an External Unit

While independent systems can provide solutions which bring comfort and avoid complexity, they showing their weaknesses in the areas of reliability, low output power (MFCs, thermoelectricity) as well as potential to cause toxicity and failing to deliver biocompatibility. Systems with external units to transfer energy continuously are therefore of interest with the increasing need of communication between IMDs and smart devices. The power can be sent through the body tissues optically, mechanically or electromagnetically, and these methods are presented and discussed in the following sections.

#### 2.2.1. Optical Charging

**Figure 6:**
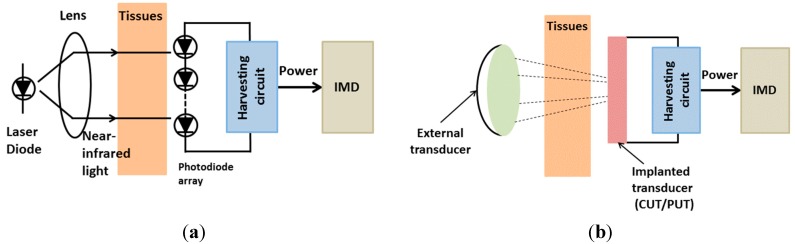
Conceptual views of: (**a**) optical charging method; and (**b**) ultrasonic transducer.

Optical charging methods utilize a photovoltaic cell integrated in the implant. The power can be transmitted through a laser diode in the near-infrared (near-IR) or infrared region and received by an array consisting of photovoltaic cells [55,117]. Light typically has low interactivity with biological tissues. Depending on the wavelength, the radiation allows to access deep inside biological tissues. The photovoltaic-cell array converts the received radiation into a current to charge/operate the IMDs (Figure 6a).

The photovoltaic cell is composed of a p-n junction of a large-band-gap semiconductor. The junction is optically charged as incident photons form electron-hole pairs, enabling electron mobility. If a load is connected, free electrons will flow through the load and then go back to the cell, where the holes are located [118]. These devices can generate power on the order of hundreds of microwatts. The photovoltaic cell array with surface area of 2.1 cm^2^ charged by a power density of 22 mW/cm for 17 min can generate a 20 μA current, which is sufficient for a pacemaker to operate for 24 h [119]. Using this approach, not only power, but information data can also be transferred using the optical link [120]. Nevertheless, during the charging period, the laser irradiation could raise the skin temperature by 1.4 °C, which may cause side effects [121]. In addition, this method remains several drawbacks, such as large size and low efficiency.

#### 2.2.2. Ultrasonic Transducer

This technology has received growing interest in the past years [56,57,58,59] due to its advantages compared with other technologies in efficiency, size and immunity to electromagnetic radiation from other devices [60]. The ultrasonic transducer is excited mechanically by an external ultrasound source using an ultrasonic transducer which can operate through capacitance mode or piezoelectric mode [122,123]. The implanted ultrasonic generator produces power from a received acoustic wave transmitted by an external unit. The incident acoustic wave is converted to electrical voltage through the capacitance change or the piezoelectric effect in cases of capacitive ultrasonic transducer (CUT) or piezoelectric ultrasonic transducer (PUT), respectively (Figure 6b). An ultrasonic power harvesting system can be fabricated using MEMS technology, therefore, the compactness makes them suitable for IMDs. However, the high voltage requirement makes CUTs unsuitable for *in vivo* operations. On the other hand, PUTs are promising as they utilize piezoelectric materials to convert the received acoustic waves into power. In 1988, Cochran *et al.* reported the first implanted ultrasonic power source which operated with an input voltage of 10–20 V at a frequency of 2.25 MHz of the external ultrasound source to excite a 5 × 5 × 0.9 mm^3^ piezo-ceramic transducer. The device could generate an output power of 1.5 mW/cm^2^ [124]. Besides transferring power, a ultrasonic system can be used to send data to an IMD [125]. Further, a piezo-ceramic transducer with a diameter of 3.5 mm implanted inside living tissues and excited by an external circular transmitter (12 mm diameter) at a frequency of 2.25 MHz was able to generate an output power up to 1.5 m·W/cm^2^ capable of driving a peripheral nerve micro-stimulator [126]. In general, the major advantage of this approach is the ease of choosing the operating wavelength. The long-wavelength ultrasounds can penetrate deeper into the human body but cover an undesirable area, contrariwise the short-wavelength waves can focus on the desirable area but they cannot penetrate the desirable depth [127]. Despite these advantages, ultrasonic power transfer still faces many challenges. In general, using ultrasonic power is considered safe and effective, but there have been some cases in which physical pain due to cavitation described as a burning feeling, was caused by the heating of the gas contained in tissue cell nuclei. This would lead to difficulty in breathing, dizziness, nausea and disorientation [127].

#### 2.2.3. Inductive Coupling

**Figure 7:**
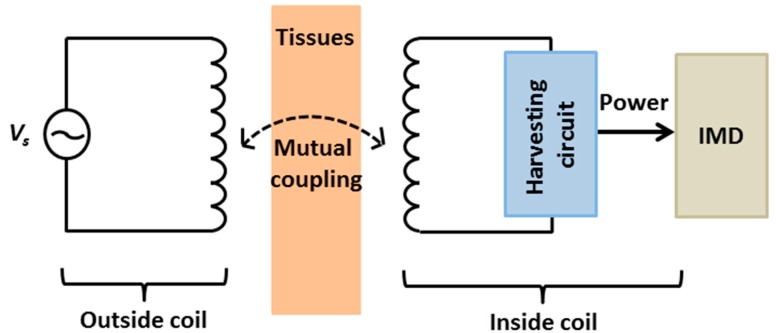
Inductive coupling overview.

It has been more than 180 years since Michael Faraday figured out power could be transferred through the air by magnetic induction. In 1914, wireless power and data transfer based on the magnetic coupling of two loops were initially reported by Tesla [128]. Recently, especially during the last decade, inductive coupling has been widely investigated to power up IMDs. The principle behind is based on a mutual inductance between two coils in which one is located outside the body while the other is integrated with the implanted device. As the external antenna transmits a varying electromagnetic signal, a voltage would be induced in the receiver coil (Figure 7). The wireless power efficiency depends on the resonance frequency (or operating frequency), distance, alignment, and coupling matching between the transmitter and the receiver coils [5,6,129,130,131,132]. In addition, the inductive technology can be exploited to transmitted data from outside to inside the body and vice-versa without using a radio-frequency (RF) transmitter or receiver with data rates up to few hundreds kilobits per second [5,6,121,133].

In their work, Parramon *et al.* developed an inductive coupling power source which could generate 19 mW at a carrier frequency of 10 MHz. This was used to power a microsystem for electromyography (EMG) recording implanted in rabbit muscle. The diameter of the inside coil, the diameter of the outside coil, and the distance between the two coil were 10 mm, 20 mm and 15 mm, respectively [134]. Another example was the use inductive coupling to power a wireless camera capsule for non-invasive visual inspection of the small bowel at a carrier frequency of 1 MHz [20]. This power source could generate up to 150 mW and the distance between the two coils was 205 mm. The size of the inside coil was 10 × 13 mm^2^. In 2004, Catrysse *et al.* presented an inductive link power source system which could deliver 50 mW over a 3 cm distance operating at 700 kHz in bench-top experiments [135]. Further, in 2007, Ghovanloo and Najafi demonstrated a system-on-chip (SOC) by combining application-specific integrated circuit (ASIC) design with off-chip components (LC tank; filters) delivering 50 mW over a 5 mm distance at 5/10 MHz frequency [136].

There are several essential factors in inductive coupling approach, such as misalignment, size of the implant antenna, unknown side effects and the limited carrier frequency due to tissue absorptions, thus low-megahertz ranges (0.3–30 MHz) have been widely used [5]. For further information about this approach, readers should refer to the Discussion section where inductive coupling is thoroughly discussed.

## 3. Discussion

### 3.1. Implantable Medical Devices: Roles and Future Expectations

With the rapid development of advanced technologies in the last decade, healthcare has been changed in several aspects. Nano- and wireless-technologies have transformed medical diagnosis, monitoring, and intervention into individualized care [6,137,138]. Definitions of self-care, e-care, mobile-health and the Internet of Things (IoTs) have emerged, changing the traditional roles of doctors and patients. People are capable of monitoring their health every day with innovative devices while large scale medical data could be assessed and diagnosed continuously by caregivers located thousands miles away. In many cases, the devices need to be inserted to stay safely and securely in the body for a period of time; thus a new expectation is raised for IMDs that the implant needs to be able to communicate with external units for real-time tracking and sensing, diagnosis and treatment. In order to achieve that, a sufficient power source becomes mandatory, no matter whether the device is active or passive.

**Figure 8:**
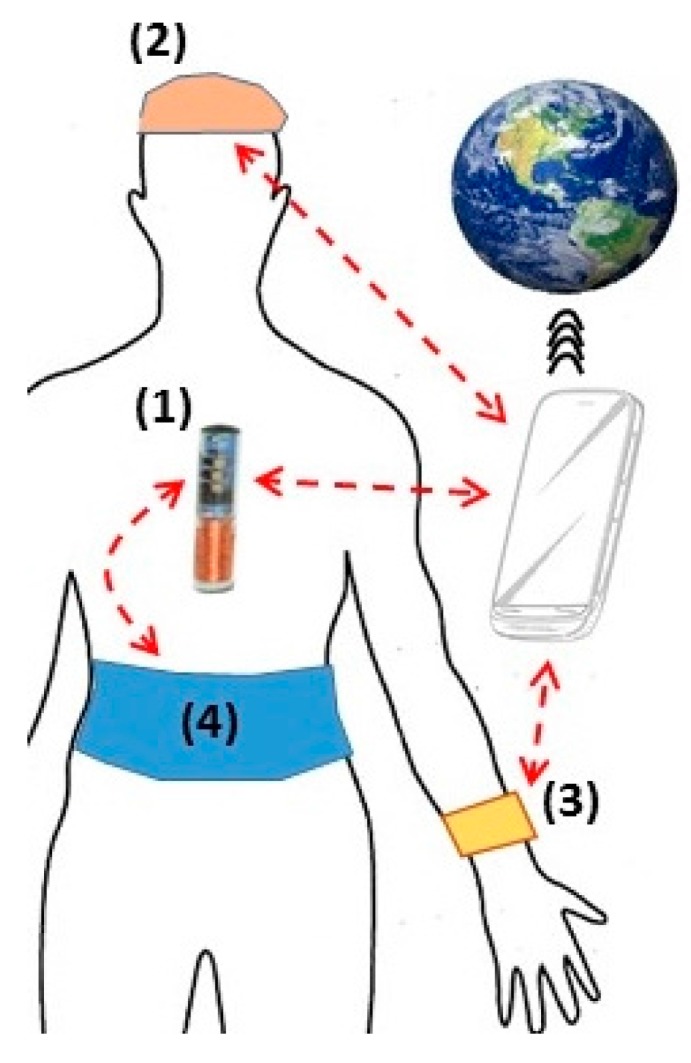
Overview of IMDs and other components. (**1**) Implant; (**2**–**4**) External devices.

In this upcoming scenario, power is needed not only for running the implant but also for feeding the high-consuming wireless communication, and this would concurrently drain out the battery of the implant and consequently reduce the lifetime of the device. A conceptual view of a future IMD is sketched in Figure 8. With these expectations, an IMD (1) can be powered from an external unit, such as a wearable belt (4) if the IMD is located in the abdomen or a hat (2) if the IMD is in the brain/head location. If there is a subcutaneous IMD in the arm/hand location, it can be powered by a wrist device (3). For wireless communications, IMDs can directly send data to a smartphone or via external units. Algorithms may be applied to filter, interpret and sort the data for diagnoses, storage or real-time examinations by distanced care-givers. Big data processes and mobile cloud systems will be needed to facilitate the infrastructure. We envision this will be the model of healthcare in the future which can significantly save time, and cost, providing efficiency and efficacy for our society. Obviously, in that dream picture, reliable micro-sensors and communication, durable power and systems as well as smart algorithms and designs, are essential. Other issues such as safety, usefulness, fashion and comfort also need to be improved in order to bring cutting-edge devices not only to patients but also the general public.

### 3.2. IMDs Power Source Approaches: Advantages and Limitations

Although there are challenges and issues that remain unsolved, IMDs have been useful and popular in healthcare applications such as medical monitoring and diagnostics. For example, a survey taken in 2009 involving 1,002,664 pacemakers and 328,027 ICDs, including new implants and replacements, found that the U.S. had the largest numbers, with 264,824 and 133,262 for pacemakers and ICDs, respectively [18]. In most cases, IMDs used non-regenerative power sources, namely batteries. To date, when safety is ensured with proper packaging, batteries still appear to be the most reliable power supply for IMDs when size, functionality and operation time are compromised. Compared with batteries, the most common issue for independent systems such as bio-fuel cells and thermoelectricity, is the low-output power while wireless powering systems add complications causing discomfort and none of the possible approaches could provide a reliability level which can be accepted in the stringent medical world.

However, sustainable sources to power chronic or life-time IMDs are still of high interest. There is another set of cases in which it is hard to access the implanted location (e.g., intravascular applications) and thus a biodegradable implant is favorable. Recent endeavors producing innovative biomaterials [139,140] have enabled the realization of those, but a batteryless solution for power using degradable materials needs to be defined. Table 1 briefly summarizes the different advantages, disadvantages, and power generation of power approaches for IMD applications.

**Table 1:** Power approaches for IMD applications.

Energy Harvesting Method	Approaches	Generated Power	References	Advantages	Disadvantages
**Independent system**	Lithium batteries	210 W·h/kg	[27]	Compatibility with flexible electronic	Size
		300 W·h/kg	[76]		Toxicity
	Bio-fuel cells	2.4 μW	[83]	Recycle materials	Lifetime
				Biocompatibility with human body	Low output power
	Nuclear batteries	50 μW	[27]	Longer service life (>15 years)	Radioactive danger
				Stable output energy	Expensive
	Thermoelectricity	5.8 µW	[91]	Unlimited lifetime	Low output power
		1 µW	[92]		
		180 μW/cm^2^	[93]		
	Piezoelectricity	2.1–69.8 W	[98]	High output power	Limited implantable locations
		0.33 μW	[104]	No additional voltage source	Biocompatibility issues
		1 W	[46]		
	Electrostatic	36 μW	[54,109]	High output power	Additional voltage source
		58 μW	[54,109]		High output impedance
		80 µW	[108]		
	Electromagnetic	40–200 μW	[112,113]	Unlimited implantable locations	Complexity in fabrication technologies
		1.1 mW	[115]		
		400 μW	[116]		
**Systems with external unit**	Optical charging	22 mW/cm	[119]	High output power	Large dimension
	Ultrasonic transducer	1.5 mW/cm^2^	[124,126]	Data transfer	Low output power
				May be used for different depths	Side effects
	Inductive coupling	19 mW	[134]	High data rate and power transmission	Limited carrier frequency due to tissue absorptions
		150 mW	[20]		
		50 mW	[135,136]	No batteries needed	Side effects
		6.15 mW	[141]		

In an expected regular IMD of the coming era, the time of m-Health and IoTs, there will be pivotal components which are highly-sensitive biosensors, low-power integrated electronic circuits, low-power and reliable wireless communication, and obviously a sufficient power source. While the recent advances in nanotechnology and materials [2] have enabled the realization of smaller and more sensitive sensors consuming less energy as well as low-power and more compact electronics, the increasing need for communication and interaction with other devices and a mobile cloud requires much higher power to operate the new-generation devices. As a result, a catch-22 without an absolute solution yet is faced. Although tremendous effort has been spent by scientists in order to investigate approaches to either exploit human energy or remotely transfer the power, the proposed solutions still cannot provide enough energy to operate smaller-and-smaller devices with more-and-more functionalities. An illustration of a pacemaker with more than 80% of volume reserved for the battery has been a real obsession and challenge for scientists working in the field, not to mention the recent call for wireless communication for m-Health applications.

Recently, numerous novel bio-sensing mechanisms using DNA [142,143,144] have been proposed implying that hybrid systems in which electronics and biological elements are merged will be possible in the coming years. Such systems would definitely require very low power but bring the highest level of sensitivity and biocompatibility as they would not be treated as foreign objects in our biological environment, not to mention minimizing biofoulings and inflammatory responses. However, as wireless data communication is still a necessity, the challenging power issue remains unsolved. Although a large number of low-power and more-effective wireless communication methodologies and protocols have been invented recently, the mechanism has remained unchanged, with wireless chips, regular amplification circuits, filters, and converters dealing with electromagnetic waves at some frequency. This would always overwhelm a compact IMD.

Besides IMDs for uses in humans, implantable devices have been widely used with animal models for biological investigations and drug screening. While the safety issues are not comparable with those of devices used for humans, most of the animal models (*i.e*., mice, zebrafish, and rats) present more challenges due to their much smaller size. Consequently, the lessons taught by overcoming the obstacles in animal models can be translated to IMDs. For instance, the recent effort in providing a wireless electrocardiogram (ECG) monitoring system to elucidate heart regeneration in zebrafish and neonatal mice [145,146] could suggest a solution for implantable devices used with humans, in which power is inductively transferred while data is sent optically.

### 3.3. Inductive Coupling: Possibilities and Challenges

Wireless powering via a resonance-based inductive link has been extensively investigated for a wide range of applications in the recent years owing to the capability to provide sufficient power, the reliability and the possibility to integrate it with other electronic components. Further, the inductive link could also be exploited for data transmission [5,6,136]. In the domain of medical devices, numerous approaches were proposed with innovations in design, materials and circuits in order to target specific applications. For IMDs, the operating frequencies are usually in the low MHz or KHz range to minimize the power absorbed by the tissue which may cause tissue heating and side effects [6,135]. A number of studies have been carried out to improve the power transfer efficiency (PTE) of regular 2-coil systems [20,23,34,147,148,149], in which coil geometry, coil dimensions, number of turns, and coil losses were examined for optimization. Recently, MEMS technology and advanced materials have enabled the fabrication of miniaturized coils on either flexible or hard substrates, which could be integrated monolithically with other electronic components. While the problem of a smaller coil resulting in less coupled energy remains unsolved, we also face with another challenge that the thin fabricated metal film of the coil (usually <1 µm) would cause an extremely low quality factor (Q), and consequently low PTE. Our group recently proposed a solution using the low-temperature co-fired ceramic (LTCC) technology to cascade the spiral coils in multiple layers (up to 10 layers in LTCC) to improve PTE (results to be published). Utilizing LTCC, improvement was achieved not only from the higher number of turns by cascading but also from the thick metal layer (>8 µm) resulting in higher Qs. Although LTCC could bring enhance PTE as well as the capability of integration with electronics as well as lab-on-a-chip applications, the hard material and the high processing temperature restrict the use with numerous IMDs.

In practical scenarios, most anatomical surfaces are highly curved and most organs are relatively mobile during daily activities, requiring IMDs to be flexible and to remain functional. However, the inductance of the flexible coil antenna would change with respect to mechanical environmental cues, thus varying the resonance frequency of the LC tank in the IMD. If the changes are significant, the PTE would be reduced drastically. This calls for the implementation of an adaptive mechanism to ensure the resonant frequencies are matched on both sides [149,150,151]. However, this may add components to the room-limited IMDs. Another practical issue is misalignment which has been widely investigated [5,35,152]. For specific IMDs attached to moving organs such as the stomach, positional and angular misalignment becomes critical. Recent investigations have shown the superiority of spiral structures for transmitter antennas to produce a large-cover beam size, minimizing misalignment issues [153]. Nonetheless, misalignment is case-dependent and unavoidable, requiring thorough calibration and investigation in a simulated environment before actual use.

Instead of using a conventional 2-antenna system, multi-input and multi-output (MIMO) systems have been investigated [154,155,156]. Obviously, as the field is continuously active, multiple IMDs can be used simultaneously, however it would lead to a complicated case as it affects the mutual inductance between any two antennas and consequently, the PTE. Further, it is also difficult to place a ‘repeater’ to improve the PTE and distance, as mentioned in [157]. The most possible case that could help is using multiple transmitters with one receiver in an IMD [156]. For example, two or more transmitters can be located around the torso to increase the power sent to IMDs. Nevertheless, it would be hard to obtain constructive superposition and field optimization in dynamic cases with the inevitable daily activities of users.

It would be improper to not mention the biological effects caused in tissues by the electromagnetic field [41,158]. Although it has been studied, attention was paid mostly to the acute effects generated by heating, while long-term health issues are concerns preventing patients and the public from accepting and using IMDs with inductive coupling.

## 4. Conclusions

The limited lifespan and biocompatibility are the most serious issues with all power approaches used in IMDs. Alternative methods have been investigated extensively to replace existing conventional battery-based systems for powering IMDs. Towards this end, this review addresses a dual goal: (1) summarize various key power approaches for IMDs and highlight the strength and weakness of each one; (2) raise concerns and discuss trends in IMDs towards future medicine and healthcare applications. All approaches for harvesting energy with independent systems and with an external unit are reviewed and discussed. The independent system approach used the body environment energy such as body temperature and body motion to generate the electrical energy to power the IMDs. These approaches are based on thermoelectric, piezoelectric, electromagnetic and electrostatic effects, providing unlimited lifetime, but the low output power and the limited implant location choices represent the major challenges. On the other hand, the power approaches using an external unit such as optical charging, ultrasonic transducer and inductive coupling allow transferring the power and information data as well as enable the ability to power the IMD in different body locations. Democritus once stated: *“**Everything existing in the universe is the fruit of chance and necessity**”.* Taking this message, we’ve fully understood the importance and significance of each approach to power IMD and highly appreciated invaluable contributions of scientists on the road towards a better life of our society.
